# Khodakarami, H., et al., Prediction of the Levodopa Challenge Test in Parkinson’s Disease Using Data from a Wrist-Worn Sensor. *Sensors* 2019, *19*, 5153

**DOI:** 10.3390/s20154167

**Published:** 2020-07-27

**Authors:** Hamid Khodakarami, Lucia Ricciardi, Maria Fiorella Contarino, Rajesh Pahwa, Kelly E. Lyons, Victor J. Geraedts, Francesca Morgante, Alison Leake, Dominic Paviour, Andrea De Angelis, Malcolm Horne

**Affiliations:** 1 Global Kinetics Pty Ltd, 31 Queens St., 3000 Melbourne, Australia; hamid.khodakarami@globalkineticscorp.com; 2 Neurosciences Research Centre, Molecular and Clinical Sciences Research Institute, St George’s University of London, Crammer Terrace, London SW18 0RE, UK; lucia.ricciardi2@gmail.com (L.R.); fmorgant@sgul.ac.uk (F.M.); a.leake@nhs.net (A.L.); dpaviour@sgul.ac.uk (D.P.); andreadeangelis.md@gmail.com (A.D.A.); 3 Department of Neurology, Leiden University Medical Center, K5-Q103 Albinusdreef 2, 2333 ZA Leiden, The Netherlands; M.F.Contarino@lumc.nl (M.F.C.); V.j.geraedts@lumc.nl (V.J.G.); 4 Department of Neurology, Haga Teaching Hospital, Els Borst-Eilersplein 275, 2545 AA The Hague, The Netherlands; 5 Parkinson’s Disease and Movement Disorder Center, University of Kansas Medical Center, 3599 Rainbow Blvd, MS 3042, Kansas City, KS 66160, USA; RPAHWA@kumc.edu (R.P.); kelly.lyons@att.net (K.E.L.); 6 Department of Clinical and Experimental Medicine, University of Messina, 98122 Messina, Italy; 7 Florey Institute of Neuroscience and Mental Health, University of Melbourne, Melbourne, VIC 3010, Australia; 8 St Vincent’s Hospital, 3065 Fitzroy, Australia

The authors wish to make the following erratum to this paper [1]:

Due to the mislabeling of the horizontal axis in Figure 4, i.e., 0 and 1 being reversed, replace the current Figure 4 (below).


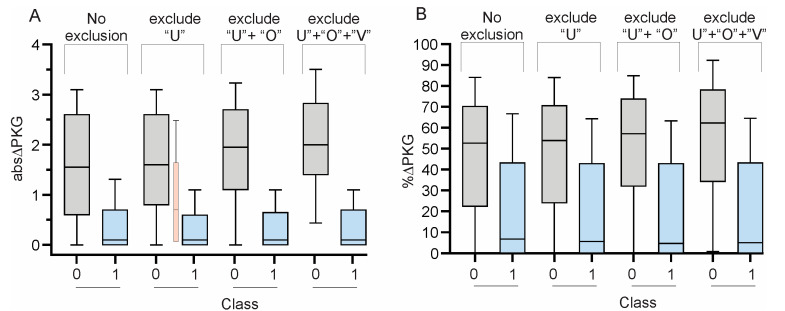


with the Figure 4 below with the correct horizontal axis labeling.


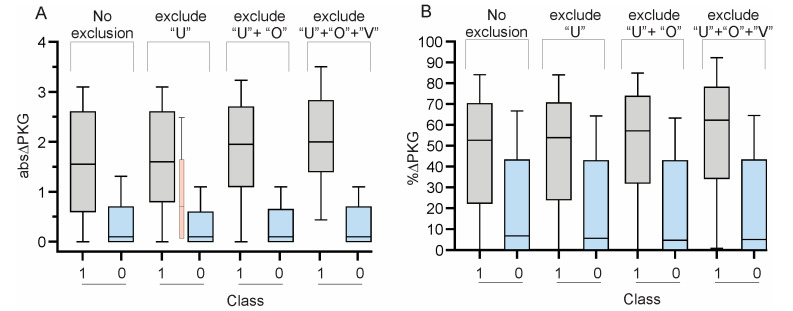


This change does not affect the scientific results. The manuscript will be updated and the original version will remain online on the article webpage, with a reference to this erratum. The authors would like to apologize for any inconvenience caused to the readers by this change.
